# Supplementary material for: A silicon photoanode protected with TiO2/stainless steel bilayer stack for solar seawater splitting
Source: Nat Commun. 2024 Apr 6;15:2970. doi: 10.1038/s41467-024-47389-z (PMC10998903; doi:10.1038/s41467-024-47389-z)
Supplement: Supplementary file 1 — Supplementary Information [file 41467_2024_47389_MOESM1_ESM.pdf]

Supplementary Information

Si photoanode protected with TiO_2/stainless steel bilayer stack for solar seawater splitting

Shixuan Zhao[1,2], Bin Liu[1,2], Kailang Li[1,2], Shujie Wang[1,2,3], Gong Zhang[1,2], Zhi-Jian Zhao[1,2,4,5], Tuo Wang[1,2,4,5*], and Jinlong Gong[1,2,3,4,5*]

[1]*School of Chemical Engineering and Technology; Key Laboratory for Green Chemical Technology of Ministry of Education, Tianjin University; Tianjin 300072, China.*

[2]*Collaborative Innovation Center of Chemical Science and Engineering (Tianjin), Tianjin 300072, China.*

[3]*Joint School of National University of Singapore and Tianjin University, International Campus of Tianjin University, Binhai New City, Fuzhou 350207, China.*

[4]*Haihe Laboratory of Sustainable Chemical Transformations, Tianjin 300192, China.*

[5]*National Industry-Education Platform of Energy Storage, Tianjin 300350, China.*

*Corresponding author. E-mail: jlgong@tju.edu.cn; wangtuo@tju.edu.cn.

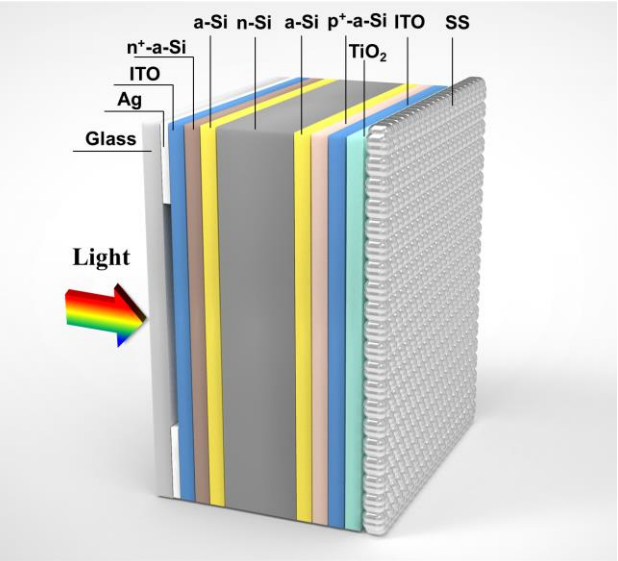

Supplementary Fig. 1 | The schematic illustration of the photoanode. The layers deposited on the photoanode are not plot according to the true proportion.

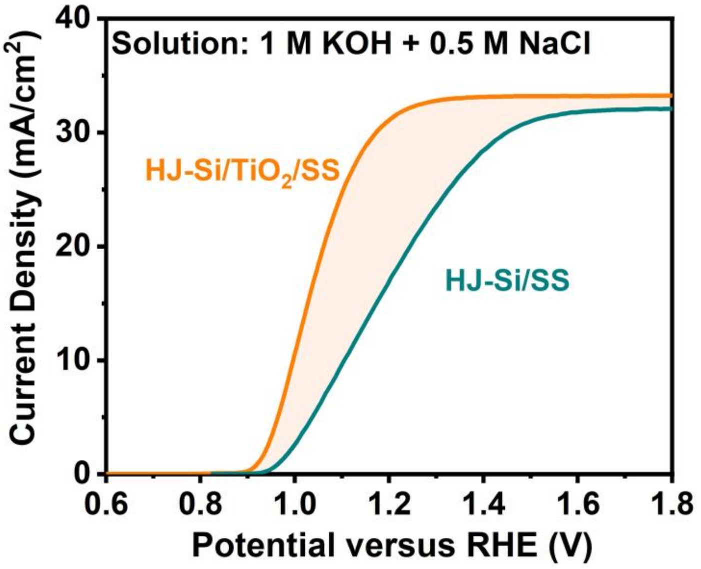

Supplementary Fig. 2 | PEC performance of different photoanodes. J-V curves of HJ-Si/TiO_2/SS and HJ-Si/SS in chloride-containing alkaline electrolytes under simulated AM 1.5G illumination.

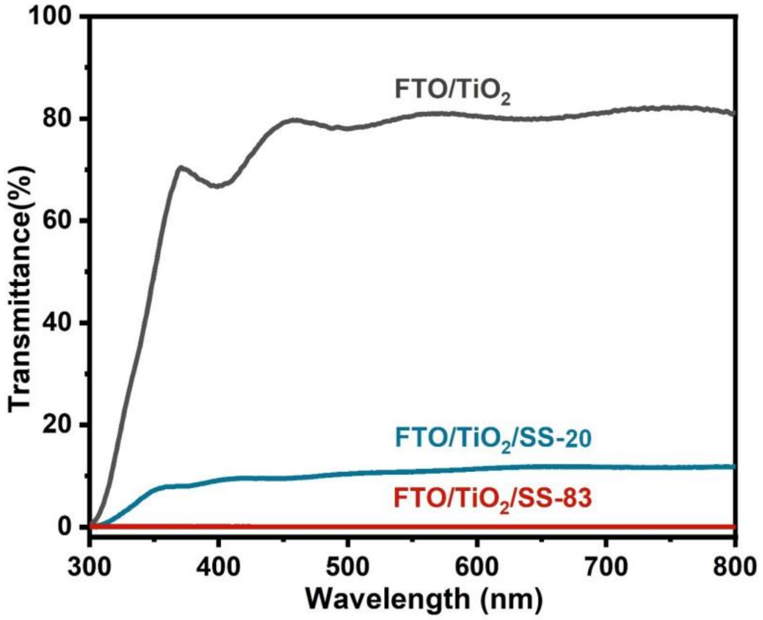

Supplementary Fig. 3 | UV spectra of electrodes. UV-vis transmission spectra of FTO/TiO_2, FTO/TiO_2/SS with different thicknesses.

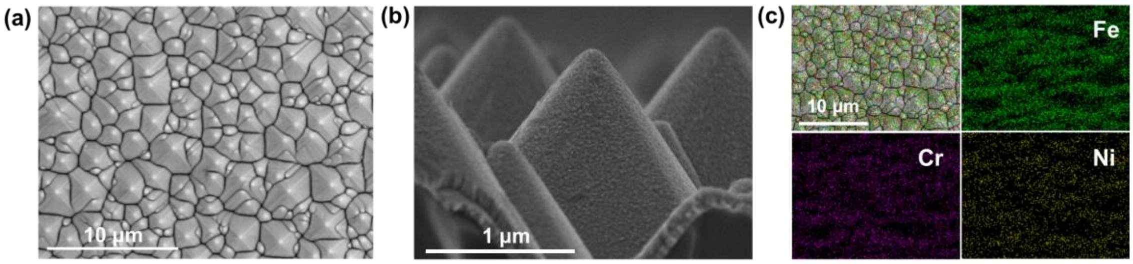

Supplementary Fig. 4 | The structure of photoanodes. (a) Top-view and (b) cross-section SEM images and (c) corresponding EDS element mappings of main elements (Ni, Fe, Cr) in pristine SS.

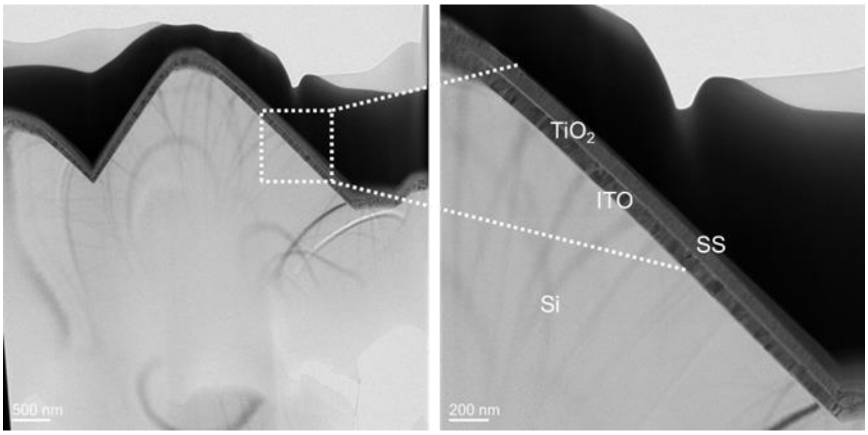

Supplementary Fig. 5 | The structural characterizations of photoanode. The cross-sectional TEM images of pristine HJ-Si/TiO_2/SS.

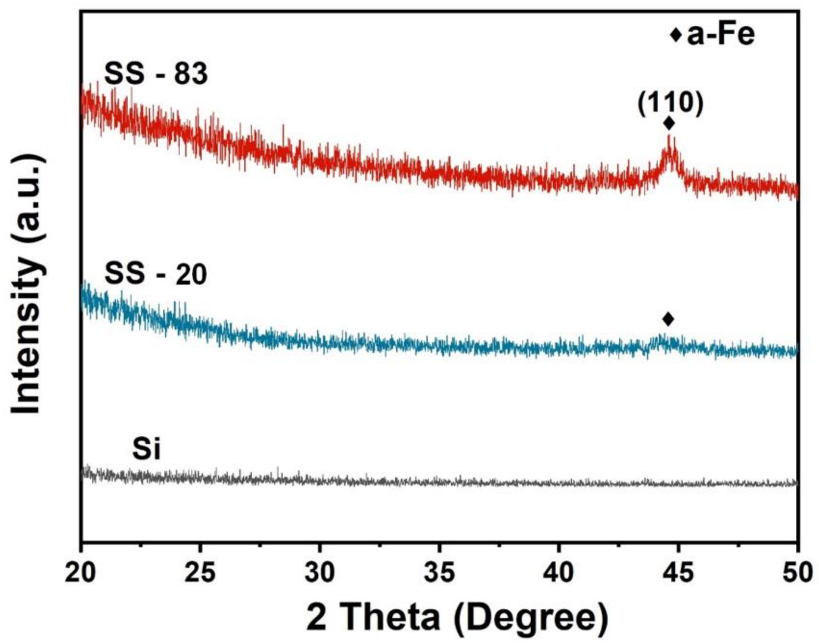

Supplementary Fig. 6 | The structural characterizations of stainless steel thin films. GIXRD patterns of pristine SS with different durations.

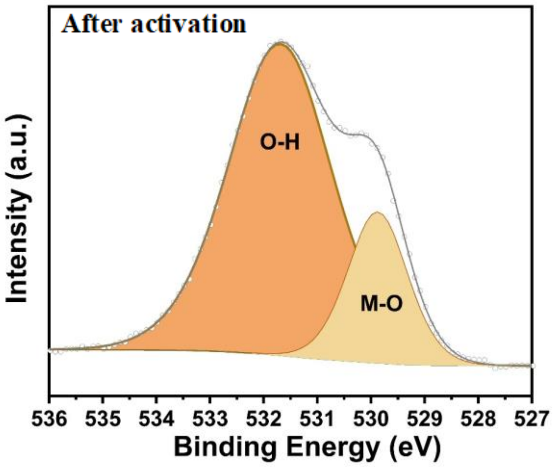

Supplementary Fig. 7 | The chemical state of O element. The high-resolution X-ray photoelectron spectroscopy of O 1s to HJ-Si/TiO_2/SS after activation.

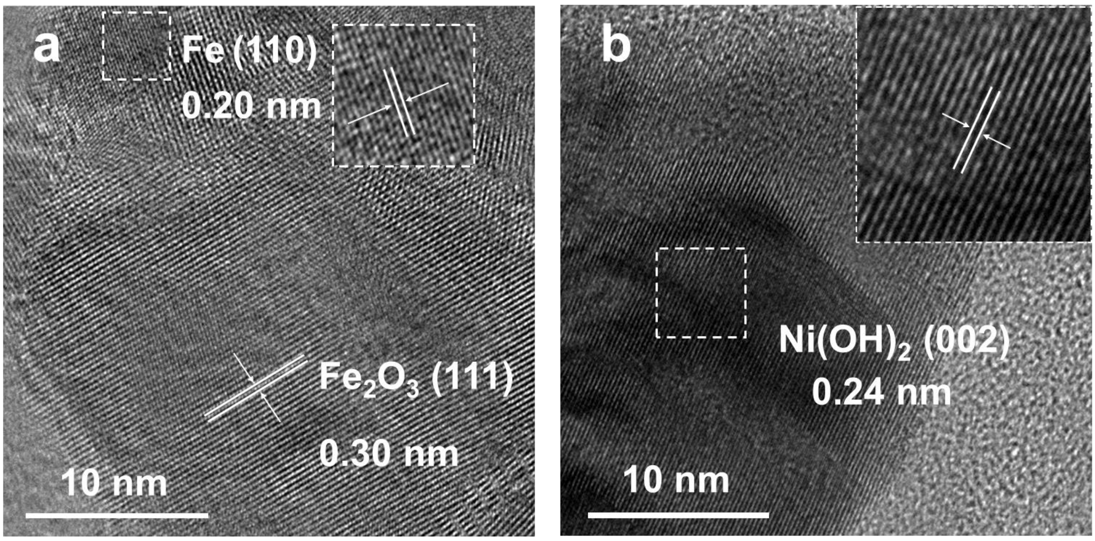

Supplementary Fig. 8 | The structure of cocatalysts before and after activation. HRTEM images of (a) pristine and (b) activated SS.

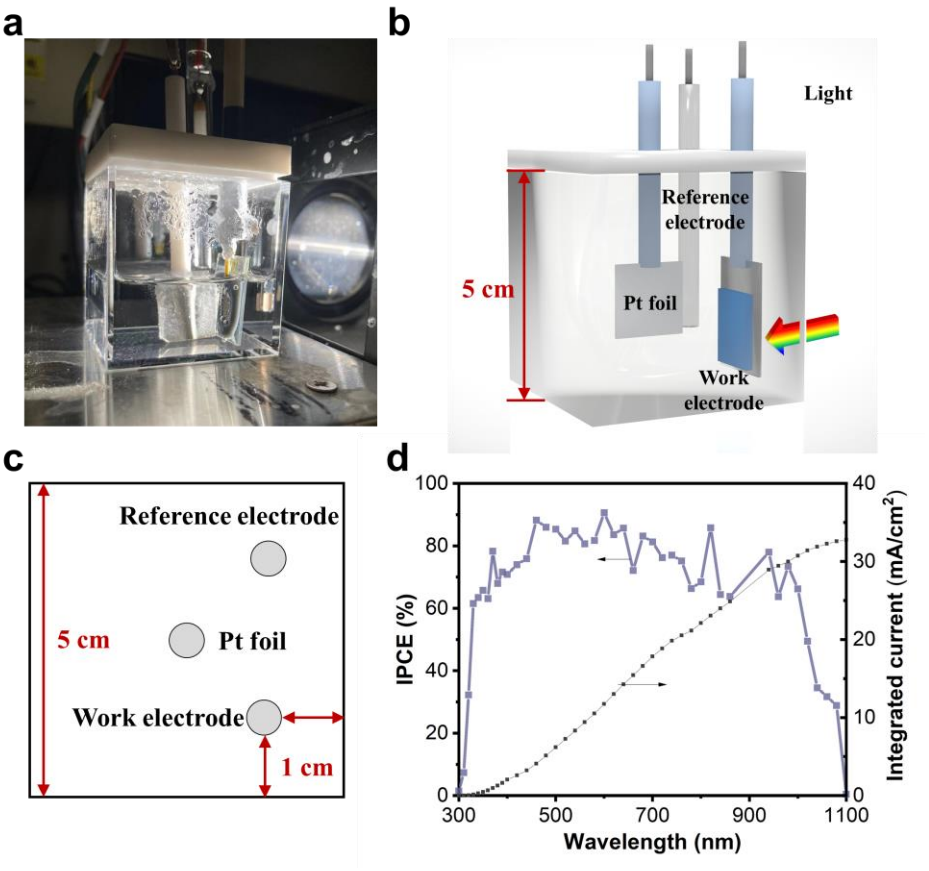

Supplementary Fig. 9 | The comprehensive structure of PEC cell and IPCE measurement. (a) The digital photograph and (b) the schematic diagram of the PEC cell. (c) The specific position of three-electrode on the lid. (d) The IPCE measured at 1.5 V vs. RHE with integrated current of photoanode against standard AM 1.5G spectrum (ASTM G173-03).

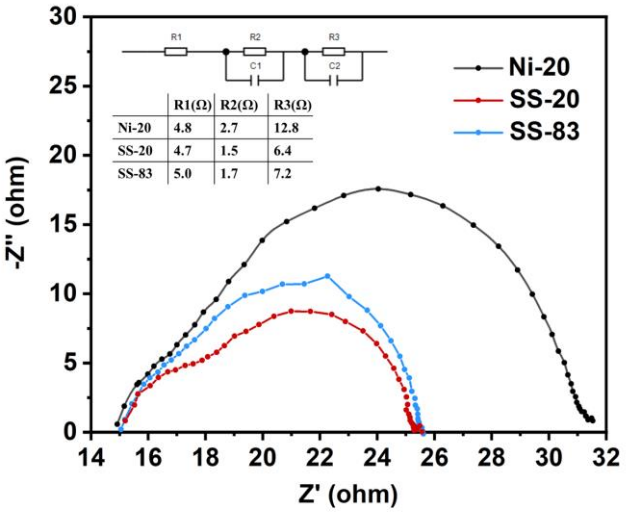

Supplementary Fig.10 | Charge transfer process. The EIS of photoanodes coated with Ni-20, SS-20 and SS-8.

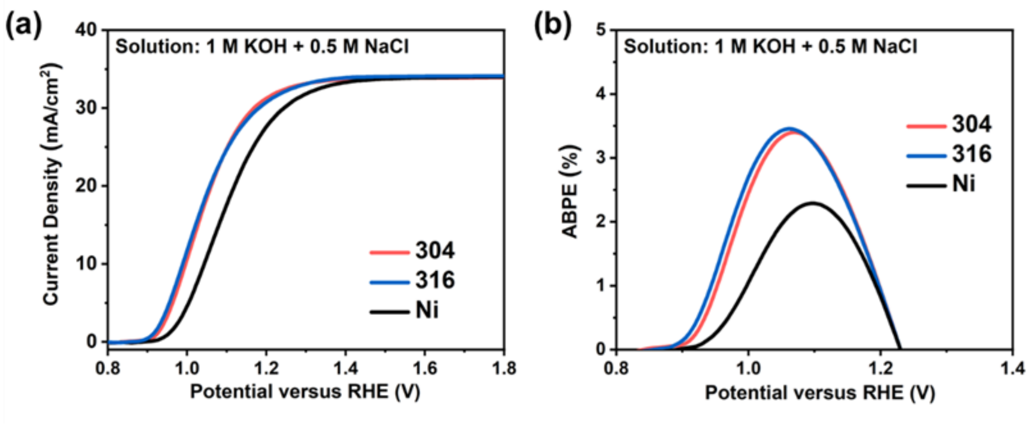

Supplementary Fig.11 | The PEC performance of photoanodes coated with different cocatalysts. (a) The current-voltage curves and (b) the corresponding ABPE curves of photoanodes coated with 304, 316 and Ni.

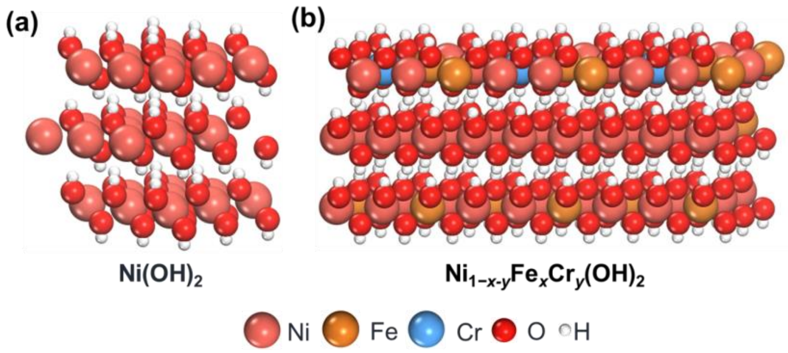

Supplementary Fig. 12 | The calculated structure of cocatalysts. The DFT models of (a) $Ni(OH)_2$ and (b) $Ni_{1-x-y}Fe_xCr_y(OH)_2$.

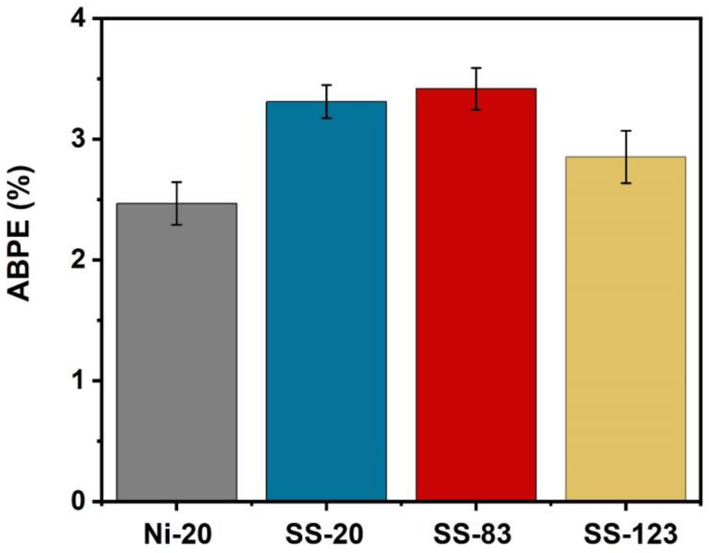

Supplementary Fig. 13 | The PEC properties of photoanodes. The ABPEs with error-bar of photoanodes decorated with various SS films and Ni in 1 M KOH + 0.5 M NaCl.

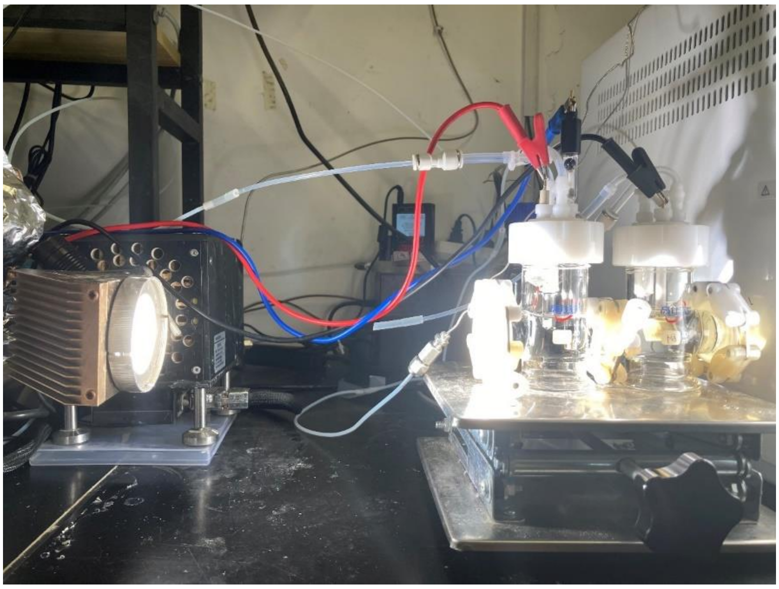

Supplementary Fig. 14 | The equipment for Faradaic efficiency. Photograph of apparatus used in the O_2 Faradaic efficiency measurement.

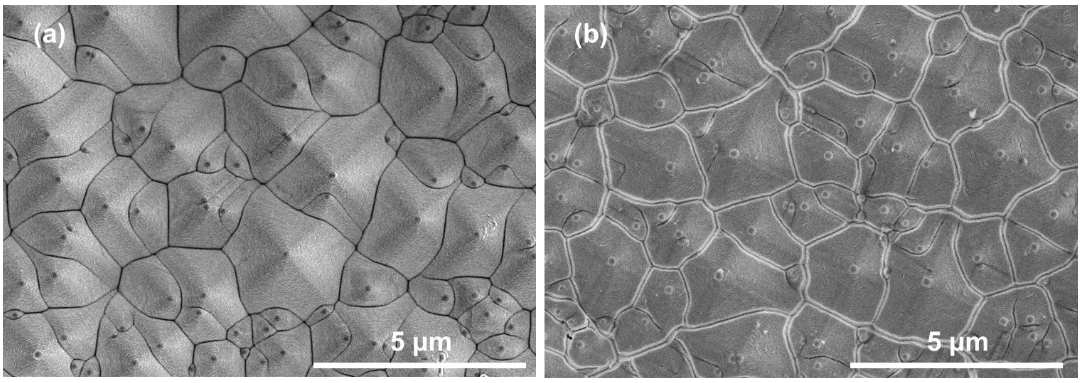

Supplementary Fig. 15 | The structure of photoanodes. SEM images of SS-83 (a) before and (b) after stability test in 1 M KOH + 0.5 M NaCl.

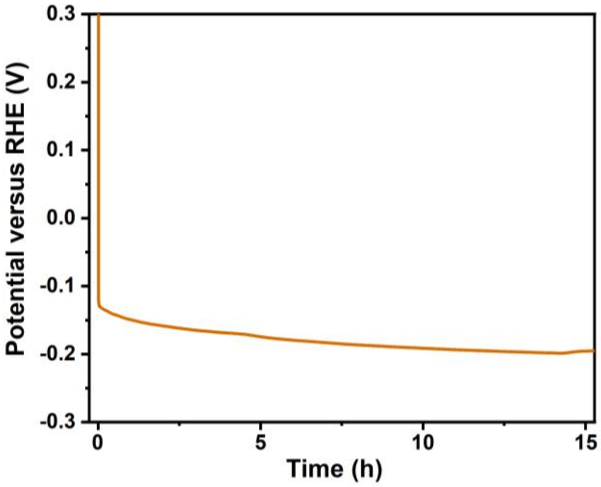

Supplementary Fig. 16 | The potential-time curve of the cathode. Cathode potential during stability in 1 M KOH + 0.5 M NaCl.

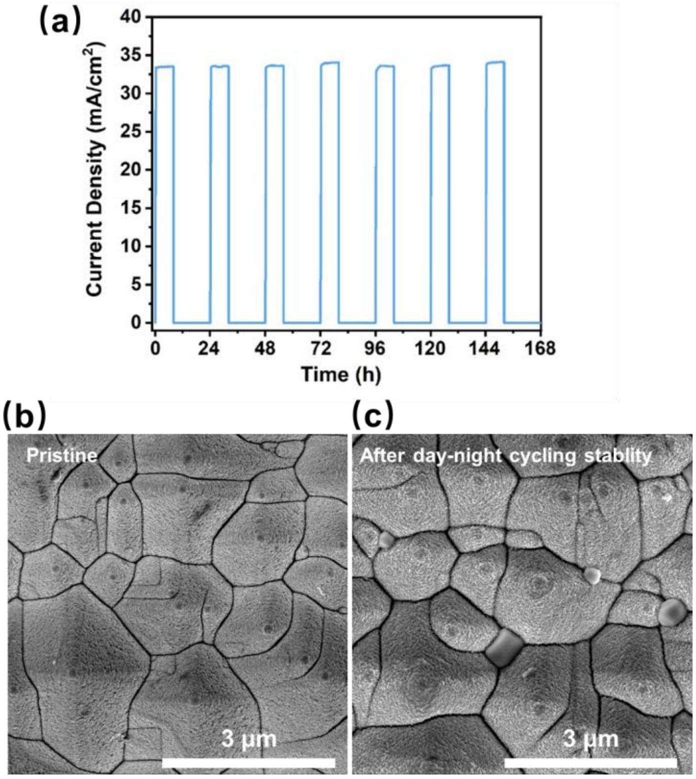

Supplementary Fig. 17 | The PEC performance for day-night run and structure of photoanode. (a) The day-night cycling stability of SS-83 in 1 M KOH + 0.5 M NaCl. The SEM images of (b) pristine and (c) photoanode after day-night cycling stability.

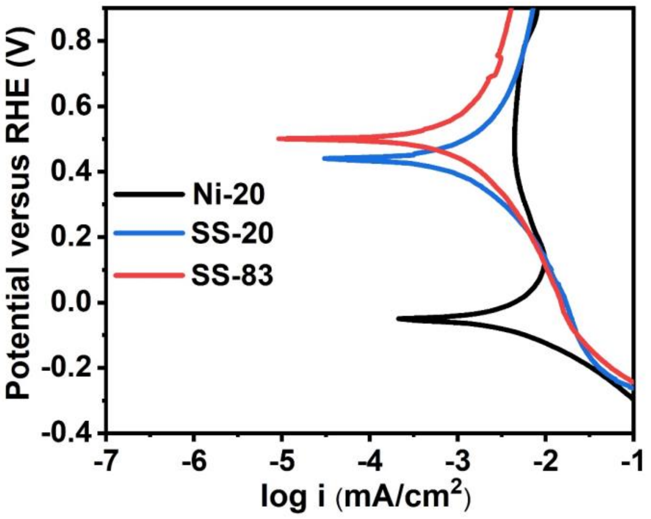

Supplementary Fig. 18 | The Tafel curves for different photoanodes. The polarization curves of photoanodes coated with SS-20, SS-83, and Ni-20 in 1 M KOH + 0.5 M NaCl.

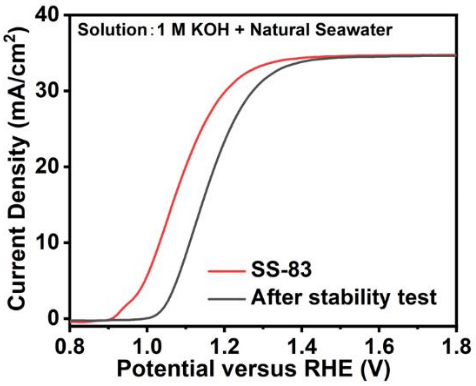

Supplementary Fig. 19 | The PEC performance of SS-83. J-V curves of SS-83 before and after stability test in 1 M KOH + Natural Seawater

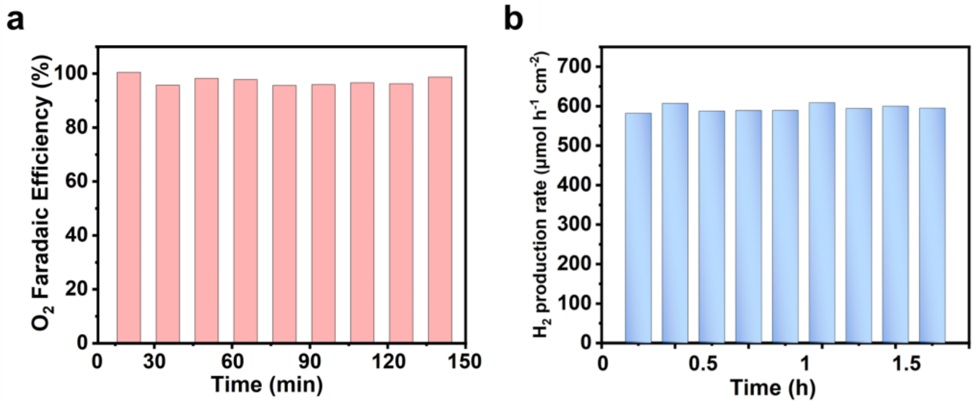

Supplementary Fig. 20 | The generation rate for different products. (a) The O_2 Faradaic efficiency and (b) the solar hydrogen production per geometric surface area in alkaline simulated seawater under simulated AM 1.5G illumination.

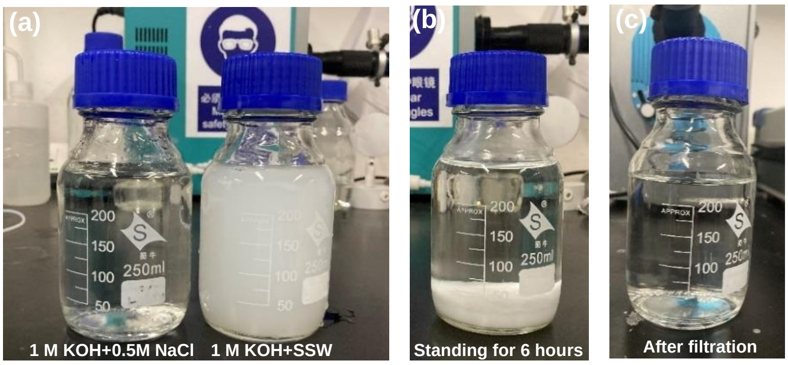

Supplementary Fig. 21 | Prepared solutions for seawater. Photographs of (a)1 M KOH plus 0.5 M NaCl (left), SSW after adding KOH (right), (b) alkaline SSW standing for 6 hours, and (c) alkaline SSW after filtration.

Supplementary Table 1 The thicknesses of metal films with various sputtering durations and targets measured by step profiler.

Thickness/nm	30 s	1 min	3 min	10 min	15 min
**Ni**	11.7	20.4	27.4		
**AISI 316L**	5.1	11.8	20.2	83	123

Supplementary Table 2 The compositions of as-prepared stainless steel films analyzed by ICP-MS

Weight%	Fe	Cr	Ni	Mn	Mo
**AISI 316L**	63-70	16-18	10-14	2	2-3
**SS**	73.8	16.7	8.1	1.3	0.06

Supplementary Table 3 The surface compositions of as-prepared and activated stainless steel films analyzed by XPS

Weight%	Fe	Cr	Ni	Mn	Mo
**AISI 316L**	63-70	16-18	10-14	2	2-3
**Pristine SS**	75.94	20.72	2.60	0.74	0
**Activated SS**	26.90	4.37	68.73	0	0

Supplementary Table 4 Reaction free energy (eV) of OER processes at U=0 and 1.23 V_{RHE} on $Ni(OH)_2$.

	**U=0 V vs. RHE**	**U=1.23 V vs. RHE**
ΔG_1	-1.39	-2.62
ΔG_2	2.24	1.01
ΔG_3	1.02	-0.21
ΔG_4	3.05	1.82

Supplementary Table 5 Reaction free energy (eV) of OER processes at U=0 and 1.23 V vs. RHE on $Ni_{1-x-y}Fe_xCr_y(OH)_2$.

	**U=0 V vs. RHE**	**U=1.23 V vs. RHE**
**ΔG_1**	-0.97	-2.2
**ΔG_2**	0.9	-0.33
**ΔG_3**	2.45	1.22
**ΔG_4**	2.58	1.31

Supplementary Table 6 The sheet resistance of SS thin films.

mΩ / sq	Ni-20	SS-20	SS-83	SS-123
**Sheet Resistance**	96.5	94.7	101.7	149.3

Supplementary Table 7 The ion concentrations in electrolytes measured by ICP-MS.

Ion concentration (μg/L)	Ni	Fe	Cr
**0.5 M NaCl + 1 M KOH**	3.7	14.5	5.1
**0.5 M NaCl + 1 M KOH after stability**	1.6	67.4	14.3
**1 M KOH + natural seawater**	1.4	4.8	4
**1 M KOH + natural seawater after stability**	1.4	40.3	8.8

The ion concentrations of electrolytes after stability were collected from SS-83 at a bias of 1.5 V vs. RHE.

Supplementary Table 8 Corrosion potentials and currents of Ni-20, SS-20, SS-83.

	**E_{corr}**	**I_{corr}**
**SS-83**	0.453	4.93×10^{-4}
**SS-20**	0.381	7.24×10^{-4}
**Ni-20**	-0.051	2.40×10^{-3}

Supplementary Table 9 Chemical Composition of Substitute Seawater

Compound	Concentration, g/L
**NaCl**	24.53
**$MgCl_2$**	5.20
**Na_2SO_4**	4.09
**$CaCl_2$**	1.16
**KCl**	0.695
**$NaHCO_3$**	0.201
**KBr**	0.101
**H_3BO_3**	0.027
**$SrCl_2$**	0.025
**NaF**	0.003

Supplementary Table 10 Summary of recent reports on various PEC seawater splitting systems.

Photoanode	Current density at 1.23 V ($mA\ cm^{-2}$)	ABPE (%)	Stable duration (h)	Total H_2 production[a] ($ml\ cm^{-2}$)	H_2 production rate[b] ($\mu mol\ h^{-1}\ cm^{-2}$)	Electrolyte / pH	
RhO_2/Mo-$BiVO_4$	2.16@ 1.0 V_{RHE}	-	4.5	4.08	40.29	Seawater/6	1
Nanostructured WO_3	4.5	-	20	37.73	83.95	Hydrochloric acid+Seawater/2	2
MoB/$BiVO_4$	4.3	-	70	126.19	80.22	Seawater/7.8	3
AgNPs + ZnFe-LDH/WO_3	1.03	0.75	6	2.59	19.22	Natural seawater/8.08	4
NiFeOx/H,1%Mo:$BiVO_4$	4.8	1.5	24	48.30	89.55	Natural seawater/8	5
Co-Pi decorated TiO_2@g-C_3N_4 nanoarrays	1.6	-	10	6.71	29.85	Natural seawater/6.4	6
Bi–BiOI shell-core microspheres modified TiO_2 nanotube arrays	1.42	0.51	<1	0.60	26.49	Artificial seawater	7
Polyaniline-graphene oxide-TiO_2 hybrid films	0.13@ 0.6 $V_{Ag/AgCl}$	-	5	0.27	2.43	Artificial seawater	8
α-Fe_2O_3/WO_3 nanorod arrays	1.02	-	<1	0.43	19.03	Natural seawater/6.8	9
**HJ-Si/TiO_2/SS-83**	**31.55**	**2.62**	**55**	**741.6**	**600**	**Alkaline natural seawater/14**	**This work**

[a]The H_2 production rate of the PEC seawater splitting systems are predicted as follows:

$$\text{The predicted amount of } H_2 = \frac{\text{The coulomb quantity of passed electrons } \times \text{FE}}{\text{The number of transferred electrons} \times \text{Faraday constant}} = \frac{\text{Current density at 1.23 V (mA cm}^{-2}\text{)} \times 100\%}{2 \times 96485}$$

The H_2 production rate of this work is measured through GC.

[b]The time used for estimating the Total H_2 production of PEC seawater splitting is the period provided by their stability tests, which represents the theoretical maximum production of H_2 for this PEC seawater splitting system.

Supplementary References

[1] Luo W, Yang Z, Li Z, et al. Solar Hydrogen Generation from Seawater with A Modified $BiVO_4$ Photoanode. *Energy Environ. Sci.* **4**, 4046-4051 (2011).

[2] Jadwiszczak M, Jakubow-Piotrowska K, Kedzierzawski P, et al. Highly Efficient Sunlight-Driven Seawater Splitting in a Photoelectrochemical Cell with Chlorine Evolved at Nanostructured WO_3 Photoanode and Hydrogen Stored as Hydride within Metallic Cathode. *Adv. Energy Mater.* **10**, 1903213 (2020).

[3] Gao RT, Guo X, Liu S, et al. Ultrastable and High-Performance Seawater-Based Photoelectrolysis System for Solar Hydrogen Generation. *Appl. Catal. B* **304**, 120883 (2022).

[4] Liu J, Xu SM, Li Y, et al. Facet Engineering of WO_3 Arrays toward Highly Efficient and Stable Photoelectrochemical Hydrogen Generation from Natural Seawater. *Appl. Catal. B* **264**: 118540 (2020).

[5] Kim JH, Hwang SM, Hwang I, et al. Seawater-Mediated Solar-to-Sodium Conversion by Bismuth Vanadate Photoanode-Photovoltaic Tandem Cell: Solar Rechargeable Seawater Battery. *iScience* **19**, 232-243 (2019).

[6] Guan X, Chowdhury FA, Pant N, et al. Efficient Unassisted Overall Photocatalytic Seawater Splitting on GaN-Based Nanowire Arrays. *J. Phys. Chem. C* **122**, 13797-13802 (2018).

[7] Li F, Dong B, Feng S. Bi Shell-BiOI Core Microspheres Modified TiO_2 Nanotube Arrays Photoanode: Improved Effect of Bi Shell on Photoelectrochemical Hydrogen Evolution in Seawater. *Int. J. Hydrogen Energy* **44**, 29986-29999 (2019).

[8] Yuan X, Xu Y, Meng H, et al. Fabrication of Ternary Polyaniline-Graphene Oxide-TiO_2 Hybrid Films with Enhanced Activity for Photoelectrocatalytic Hydrogen Production. *Sep. Purif. Technol.* **193**: 358-367 (2018).

[9] Li Y, Feng J, Li H, et al. Photoelectrochemical Splitting of Natural Seawater with α-Fe_2O_3/WO_3 Nanorod Arrays. *Int. J. Hydrogen Energy* **41**, 4096-4105 (2016).
